# A Novel Cocrystal of Daidzein with Piperazine to Optimize the Solubility, Permeability and Bioavailability of Daidzein

**DOI:** 10.3390/molecules29081710

**Published:** 2024-04-10

**Authors:** Zhipeng Wang, Shuang Li, Qi Li, Wenwen Wang, Meiru Liu, Shiying Yang, Li Zhang, Dezhi Yang, Guanhua Du, Yang Lu

**Affiliations:** 1 Beijing City Key Laboratory of Polymorphic Drugs, Center of Pharmaceutical Polymorphs, Institute of Materia Medica, Chinese Academy of Medical Sciences and Peking Union Medical College, Beijing 100050, China; zp091215@hotmail.com (Z.W.); lishuang990515@163.com (S.L.); 13544580198@foxmail.com (Q.L.); wang987214342@163.com (W.W.); mrliu1209@163.com (M.L.); ysy@imm.ac.cn (S.Y.); 2 Beijing City Key Laboratory of Drug Target and Screening Research, National Center for Pharmaceutical Screening, Institute of Materia Medica, Chinese Academy of Medical Sciences and Peking Union Medical College, Beijing 100050, China; dugh@imm.ac.cn

**Keywords:** daidzein, piperazine, cocrystal strategy, solubility, permeability, bioavailability

## Abstract

It is well known that daidzein has various significant medicinal values and health benefits, such as anti-oxidant, anti-inflammatory, anti-cancer, anti-diabetic, cholesterol lowering, neuroprotective, cardioprotective and so on. To our disappointment, poor solubility, low permeability and inferior bioavailability seriously limit its clinical application and market development. To optimize the solubility, permeability and bioavailability of daidzein, the cocrystal of daidzein and piperazine was prepared through a scientific and reasonable design, which was thoroughly characterized by single-crystal X-ray diffraction, powder X-ray diffraction, Fourier transform infrared spectroscopy, differential scanning calorimetry and thermogravimetric analysis. Combining single-crystal X-ray diffraction analysis with theoretical calculation, detailed structural information on the cocrystal was clarified and validated. In addition, a series of evaluations on the pharmacogenetic properties of the cocrystal were investigated. The results indicated that the cocrystal of daidzein and piperazine possessed the favorable stability, increased solubility, improved permeability and optimized bioavailability of daidzein. Compared with the parent drug, the formation of cocrystal, respectively, resulted in 3.9-, 3.1-, 4.9- and 60.8-fold enhancement in the solubility in four different media, 4.8-fold elevation in the permeability and 3.2-fold in the bioavailability of daidzein. Targeting the pharmaceutical defects of daidzein, the surprising elevation in the solubility, permeability and bioavailability of daidzein was realized by a clever cocrystal strategy, which not only devoted assistance to the market development and clinical application of daidzein but also paved a new path to address the drug-forming defects of insoluble drugs.

## 1. Introduction

**Figure 1:**
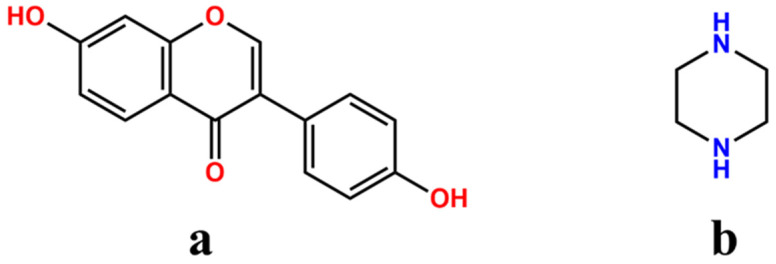
Chemical structures of daidzein (**a**) and piperazine (**b**).

As a polyphenolic isoflavone mainly derived from leguminous plants, daidzein (DAI) (Figure 1a) is reported to have significant medicinal value and health benefits [1]. With numerous potential health-promoting bioactivities, including anti-oxidant, anti-inflammatory, anti-diabetic, cholesterol-lowering, neuroprotective, cardioprotective and estrogenic effects, DAI has great potential in the treatment of cancer, diabetes, cardiovascular disease, nerve injury, osteoporosis and menopausal syndrome in women [2,3,4,5]. Considering its multiple pharmacological effects and low toxicity [6], the market development and clinical application value of DAI attracts the wide attention of pharmaceutical researchers. Unfortunately, disappointing physicochemical properties, including poor solubility, low partition coefficient and high intestine and hepatic metabolism result in inferior bioavailability [7,8], which hinders its therapeutic effects and limits the potential applications for clinical.

To address the dilemma of poor water solubility and low bioavailability of DAI for drug formation, various attempts, including cyclodextrin inclusion complex [9], liposome [10], nanoparticles [11], chitosan microspheres [12] and emulsions [13], are taken into practice. However, there are some deficiencies, such as undefined kinetics and neurotoxicity in nanoparticles, gastric toxicity and huge bulk of cyclodextrins and stability flaws of proteins, in chitosan microspheres in these approaches [14], which bring about a series of emerging issues for the drug development and clinical application of DAI. Compared with these strategies, cocrystal technology is a green, simple and efficient route to optimize the physiochemical and biopharmaceutical properties of drugs without generating adducts, intermediates or any waste products. Nowadays, it is demonstrated that cocrystallization can overcome several issues in the aspects of optical properties, mechanical properties, hygroscopicity, stability, solubility, permeability and bioavailability [15,16,17,18,19]. In addition, cocrystal technology also plays a significant role in the pharmaceutical theater to reduce drug adverse reactions, enhance drug therapeutic effects and create new patents and intellectual property [20,21]. Moreover, several pharmaceutical cocrystal products have acquired extreme acceptance and popularity in the market, and clinical studies also provide great evidence for the utility and great potential of cocrystal technology [21,22].

To the best of our knowledge, an excellent pharmaceutical cocrystal product benefits from scientific and reasonable cocrystal design. The phenolic hydroxyl groups of DAI give it a chance to form cocrystals via a variety of supramolecular synthons [23]. In the process of cocrystal design, intermolecular interaction complementarity and supramolecular synthon hierarchy are the primary considerations. Based on the weak acidity of DAI and the supramolecular hetero-synthons OH⋯NH, piperazine (PPZ) (Figure 1b) is selected to serve as a suitable cocrystal former coupled with the superior solubility and high safety of PPZ [24]. In addition, the piperazine ring is considered an important class of nitrogen heterocycles that can effectively regulate the lipid–water partition coefficient and acid–base equilibrium constants of drugs, which may have positive influences on improving the druggability of DAI [25]. What is more, a series of the literature on the cocrystals with PPZ demonstrates that the introduction of PPZ can significantly optimize the solubility and bioavailability of active pharmaceutical ingredients (APIs) [24,26,27].

Until now, there have been some studies on the cocrystal of DAI reported [14,28,29]. Among these studies, there is no single crystal successfully grown for its structure confirmation apart from the daidzein-4,4′-bipyridine cocrystal. Except for obtaining a single crystal of the DAI-PPZ cocrystal, an investigation on the DAI-PPZ cocrystal was launched systematically and comprehensively from the aspects of preparation, characterization, theoretical computation, structural analysis, stability, solubility, dissolution, permeability and bioavailability evaluation. Owing to the theoretical calculations, the intermolecular forces forming cocrystals were clarified, and the types of hydrogen bonds involved in the generation of cocrystals were identified. Combined with the SCXRD analysis, the formation of DAI-PPZ cocrystal was further confirmed and the mechanism of cocrystal formation was elucidated in depth. In addition, the surprising results indicated that the cocrystal between DAI and PPZ in this study achieved a more significant performance in enhancing the solubility, permeability and bioavailability of DAI when compared with previous studies. Based on a clever and rational cocrystal strategy, the pharmaceutical defects of DAI, including solubility and bioavailability, were overcome, which not only contributed assistance to improving the pharmacological activity of DAI but also broke a new avenue to solve the problem related to the poor drug formation of natural products.

## 2. Results and Discussion

### 2.1. Crystal Structure Analysis

As the most authoritative characterization tool for the identification of cocrystals, SCXRD analysis can provide the three-dimensional structure of a cocrystal and various basic information for further theoretical computation and the analysis of different forces that hold the crystal structure together. To the best of our knowledge, the hydrogen bond is considered a common intermolecular force that plays an important role in the process of cocrystal formation. Widely used to analyze the capabilities of molecules as hydrogen bond donors and acceptors, MEPS analysis is popular with a broad range of researchers. In this study, SCXRD analysis and MEPS calculations were skillfully combined to elucidate the formation mechanism of the DAI-PPZ cocrystal in depth.

**Figure 2:**
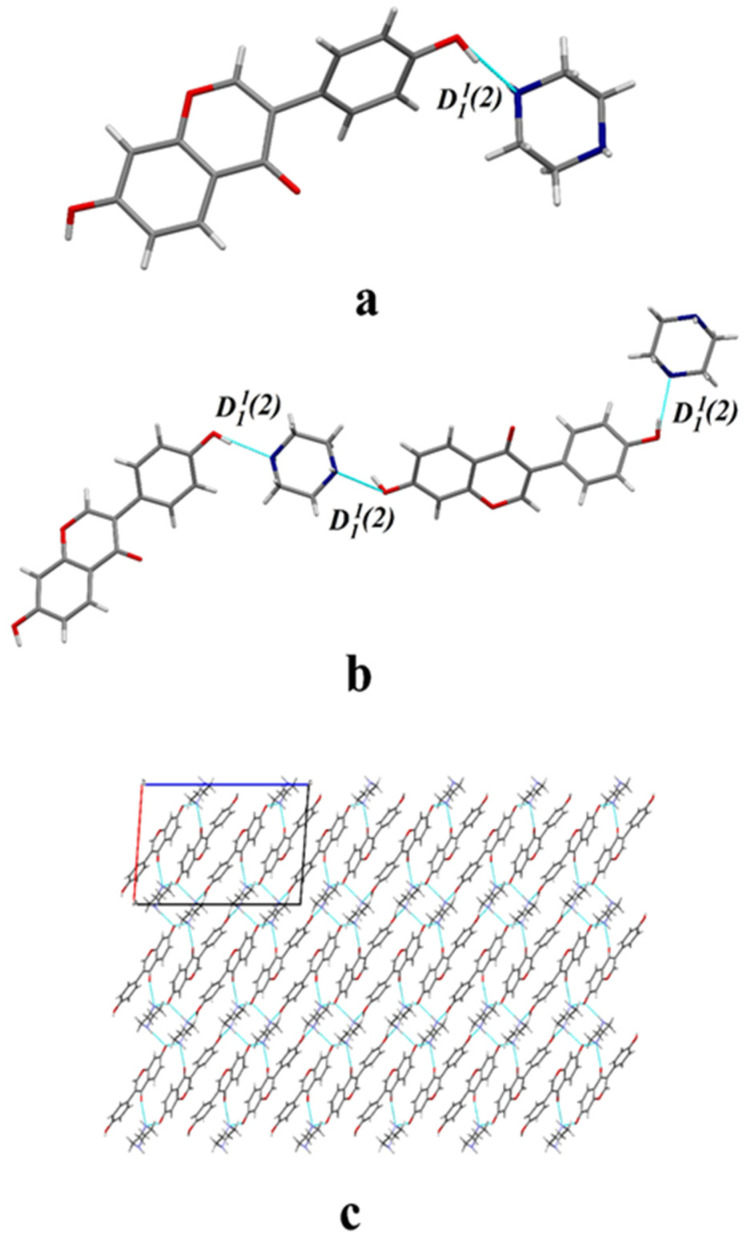
Crystal structure of the DAI-PPZ cocrystal: (**a**) asymmetric unit, (**b**) a chain structure, (**c**) a neat laminar structure viewed from the b axis.

**Figure 3:**
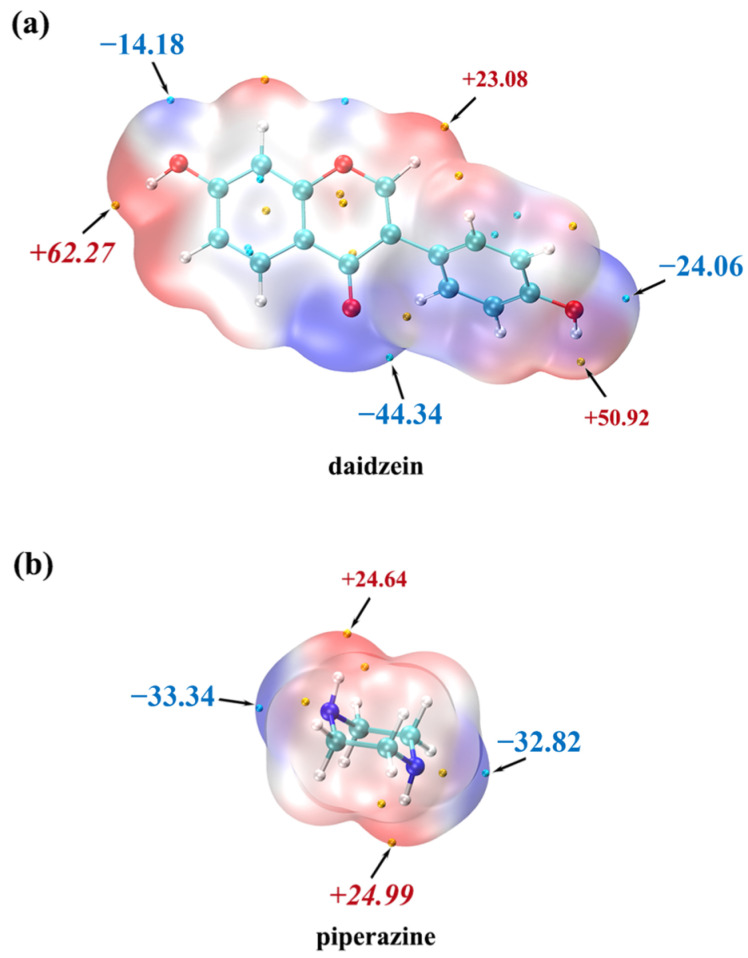
The results of MEP calculations on the cocrystal of daidzein (**a**) and piperazine (**b**).

According to the information provided in Table 1, the crystal structure of DAI-PPZ is classified into the P21/C space group of the monoclinic crystal system. It is obviously found that there are one DAI and one PPZ in the asymmetric unit from Figure 2a. In Figure 2b, O–H⋯N hydrogen bonds mainly participate in the formation of DAI-PPZ cocrystal. DAI is linked with PPZ to form a chain structure through O3–H3⋯N1P (2.557 Å) and O4–H4⋯N2P (2.738 Å) in a head-to-tail sequence. It is depicted in Figure 2c that a neat laminar structure is stacked by the formed chain structure under the action of these two hydrogen bonds, namely N1P–H1P⋯O2 (2.919 Å) and N2P–H2P⋯O3. To the best of our knowledge, the main interaction sites in the cocrystal should appear pairwise in the minima and maxima of the MEPS first, followed by the secondary ones [30]. It can be found in Figure 3 that the site of O3 (62.27 kcal mol^−1^) is the first global maxima site of DAI, and the site of N1P (−33.34 kcal mol^−1^) is the first global minima site of PPZ. Likewise, the site of O4 (50.92 kcal mol^−1^) is the secondary global maxima site of DAI and the site of N2P (−32.82 kcal mol^−1^) is the secondary global minima site of PPZ. With the help of the MEPS analysis results, it is predicted that the interaction of O3–H3⋯N1P is strongest, and the interaction of O4–H4⋯N2P is inferior, which is further confirmed by their individual bond length (O3–H3⋯N1P is 2.919 Å and O4–H4⋯N2P is 3.092 Å). Based on the above analysis, it can be concluded that the hydrogen bonds, including O3–H3⋯N1P and O4–H4⋯N2P, are the two main participators in the formation of DAI-PPZ. In addition, relative crystallographic and hydrogen bond information on the DAI-PPZ cocrystal structure are, respectively, presented in Table 1 and Table 2.

### 2.2. PXRD Analysis

**Figure 4:**
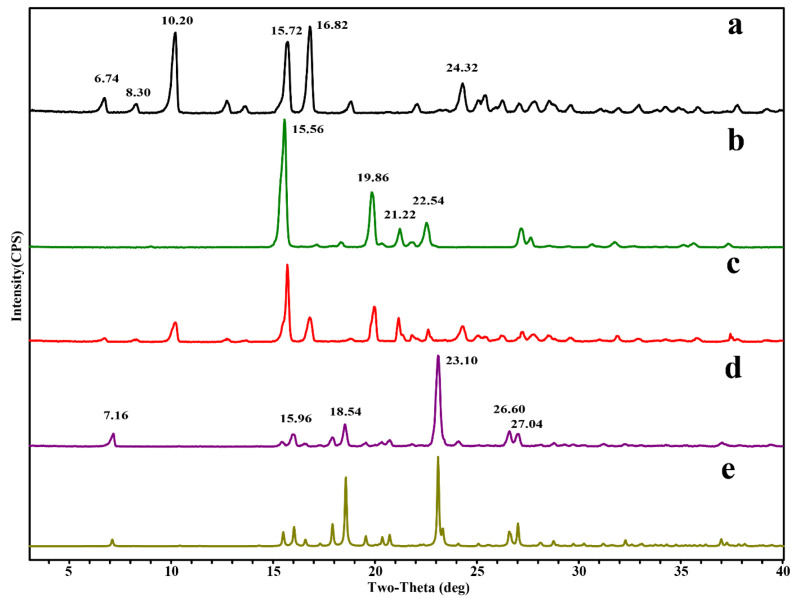
The PXRD pattern of DAI (a), PPZ (b), the physical mixture of DAI and PPZ (c), the prepared DAI-PPZ cocrystal (d), the simulated DAI-PPZ cocrystal calculated from the SCXRD data (e).

Generally speaking, its physical phase will undergo significant changes along with the generation of cocrystals. In this study, the characteristic crystalline peaks attributed to DAI appeared at 2θ values of 6.74°, 8.30°, 10.20°, 12.74°, 13.66°, 15.72°, 16.82°, 22.06° and 24.32°. At the same time, characteristic crystalline peaks attributed to PPZ appeared at 15.56°, 19.86°, 21.22°, 22.54°, 27.20°, 27.66° and 31.76°. Differing from the physical mixture of DAI and PPZ, there was a unique PXRD pattern in the PXRD pattern of DAI-PPZ cocrystal, whose new peaks exhibited at 2θ values of 7.16°, 15.44°, 15.96°, 16.54°, 17.26°, 17.90°, 18.54°, 19.56°, 20.34°, 23.10°, 26.60° and 27.04° (Figure 4). According to the results, the characteristic peaks of both DAI and PPZ disappeared, and a series of new peaks emerged in the PXRD pattern of the DAI-PPZ cocrystal. It was predicted that a new crystalline was generated. This prediction was further confirmed by the excellent match between the experimental pattern and the simulated powder patterns calculated from the SCXRD data.

### 2.3. IR Analysis

**Figure 5:**
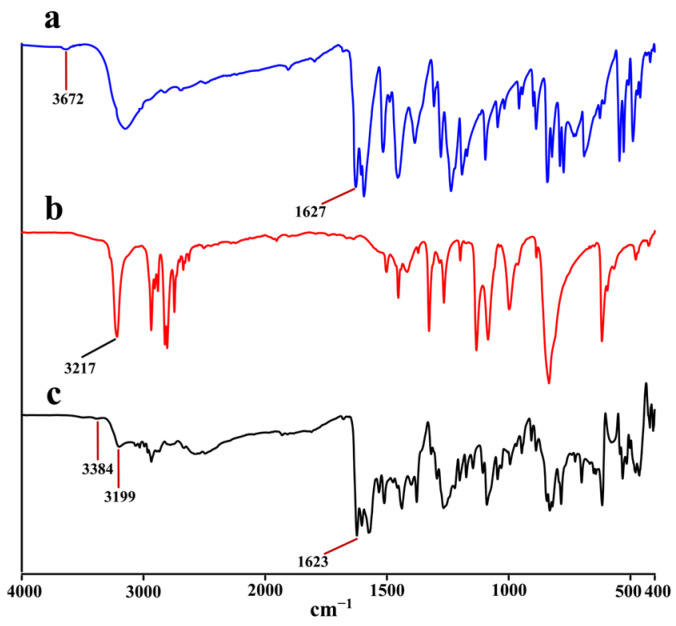
The results of IR analysis of DAI (a), PPZ (b) and the DAI-PPZ cocrystal (c).

In this study, the changes in the noncovalent interactions accompanied by the formation of cocrystals were investigated by recording and comparing the FT-IR spectra of DAI, PPZ and DAI-PPZ. The results of SCXRD demonstrated that the hydrogen bond was the main participator in the generation of cocrystal, which was further corroborated by the fact that the IR spectra of the cocrystal were significantly distinct from DAI and PPZ. It is known to us that the formation of hydrogen bonds can result in the IR wavenumbers shifting toward shorter ones. In Figure 5a, the free O–H stretching absorption peak appeared at 3672 cm^−1^, and the C=O stretching absorption peaks emerged at 1627 cm^−1^. In Figure 5b, the free N−H stretching absorption peak appeared at 3217 cm^−1^. Obviously, it can be found in Figure 5c that a blue shift occurred in the band attributed to ν_O–H_ (3672 cm^−1^ in the IR spectrum of DAI vs. 3384 cm^−1^ in the IR spectrum of DAI-PPZ) and the band attributed to ν_N–H_ (3217 cm^−1^ in the IR spectrum of PPZ vs. 3199 cm^−1^ in the IR spectrum of DAI-PPZ). Additionally, the ν_C=O_ stretching band at 1627 cm^−1^ in the spectra of DAI also blue shifted to the band at 1623 cm^−1^ in the DAI–PPZ spectrum. These findings further provided some significant information to prove the occurrence of the intermolecular hydrogen bonds between DAI and PPZ, including O–H⋯N and C=O⋯H–N.

### 2.4. Thermal Analysis

**Figure 6:**
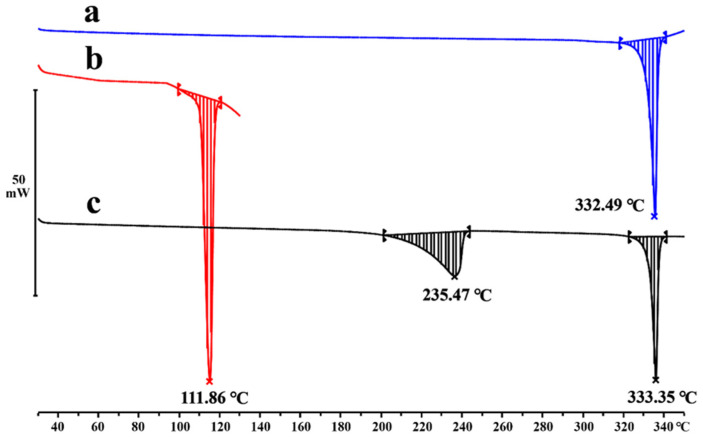
The DSC plot of DAI (a), PPZ (b) and the DAI-PPZ cocrystal (c).

**Figure 7:**
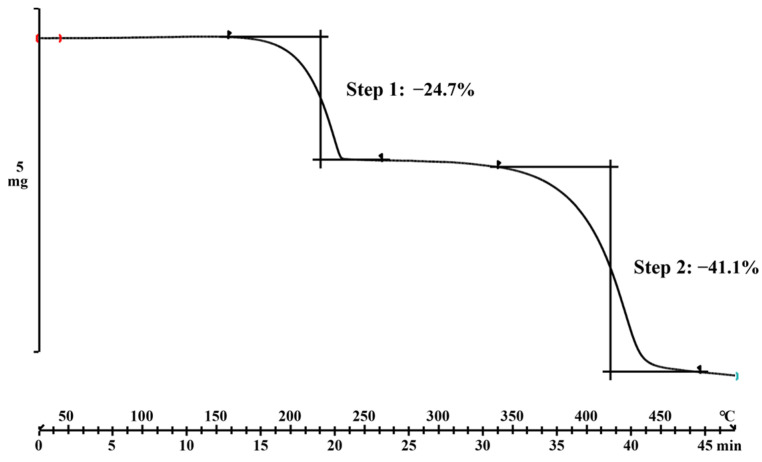
The TG plot of the DAI-PPZ cocrystal.

Combining with DSC and TG, the thermodynamic behavior of materials can be revealed and evaluated. Differing from the peaks of DAI (332.49 °C) and PPZ (111.86 °C), the endothermic peak of the DAI-PPZ cocrystal emerged at 235.47 °C (Figure 6), which suggested the generation of a new solid phase. To our surprise, an interesting phenomenon was that two endothermic peaks at 235.47 °C and 333.35 °C occurred in the DSC plot of the DAI-PPZ cocrystal. It was worth emphasizing that the sharp endothermic peak at 333.35 °C was quite near to the melting point of DAI (332.49 °C). In addition, the results of TG (Figure 7) indicated that the first weight loss of 24.7% from 160 to 254 °C was reckoned as the loss of the PPZ molecule, whose theoretical weight percentage was 25.3%. To our knowledge, PPZ has a low boiling point of about 146.0 °C. Based on this evidence, it could be predicted that the cocrystal decomposed at 235.47 °C, and PPZ was sublimated with the elevation of temperature, whose phenomenon was similar to the thermodynamic characteristics of solvent compounds and reported in previous studies [25,31,32]. Additionally, it could be found that there was no mass loss to be observed in the test temperature range below the melting point of the DAI-PPZ cocrystal, which indicated that the cocrystal of DAI-PPZ did not contain water or solvents.

### 2.5. Stability Study

**Figure 8:**
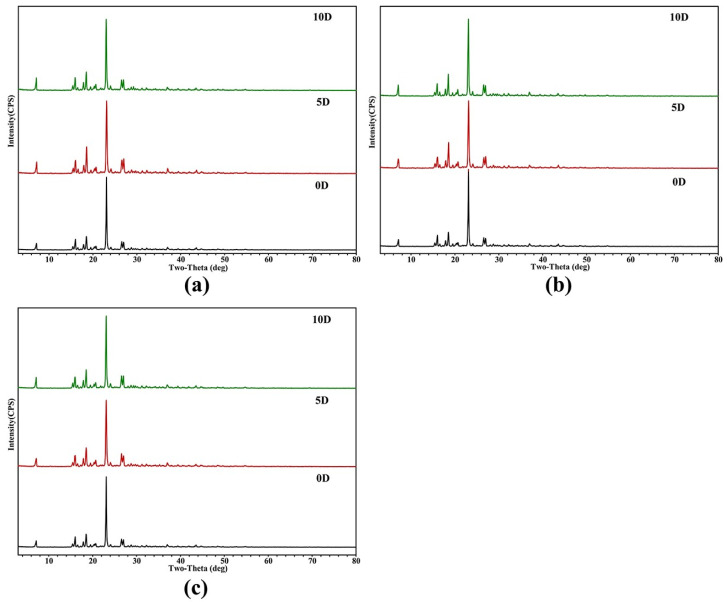
PXRD comparisons on the stability study of the cocrystal between daidzein and piperazine ((**a**) under high temperature, (**b**) high humidity and (**c**) under illuminated environments).

Considered an integral property of drugs, the evaluation of stability is essential. On days 0, 5 and 10, the stability of the DAI-PPZ cocrystal in high temperature, high-humidity and illuminated environments was investigated in this study. According to the evaluation results (Figure 8), the PXRD patterns of the cocrystal on days 5 and 10 had a superior match with its PXRD patterns on day 0 under high-temperature, high-humidity and illuminated environments, which indicated that the cocrystal of DAI-PPZ owned good stability under these three extreme conditions.

### 2.6. Solubility and Powder Dissolution In Vitro

**Figure 9:**
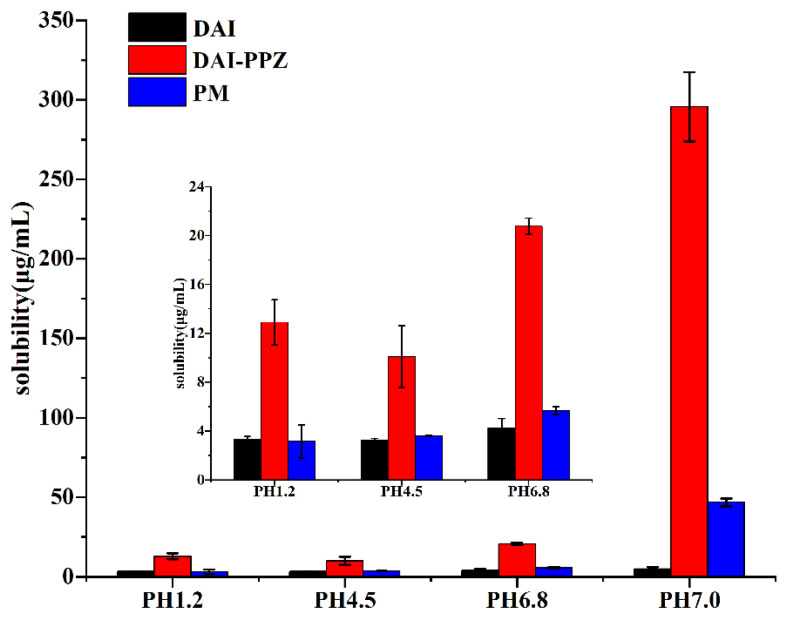
Comparison between the equilibrium solubilities of the pure DAI, the DAI-PPZ cocrystal and the physical mixture of DAI and PPZ in four different media (pH = 1.2, pH = 4.5, pH = 6.8 and pH = 7.0).

With a planar spatial structure, the molecule of DAI is tightly packed. In addition, two phenolic hydroxyl groups at positions 7 and 4′ in the structure of DAI can form intermolecular hydrogen bonds. Due to the arrangement of the lattice and the formation of hydrogen bonds, the intermolecular force of DAI is increased, which leads to the poor solubility of DAI. According to the previous study, low solubility is closely related to poor bioabsorption [33]. To optimize the solubility of DAI, cocrystal technology was employed in this study. Based on the results of the equilibrium solubility study (Figure 9), the equilibrium solubility of DAI in four different mediums (pH 1.2, pH 4.5, pH 6.8 and pH 7.0) was, respectively, 3.31 ± 0.24, 3.29 ± 0.12, 4.27 ± 0.76 and 4.86 ± 1.25 μg/mL. The equilibrium solubility of DAI in the physical mixture was individually 3.16 ± 1.35, 3.62 ± 0.07, 5.69 ± 0.31 and 46.80 ± 2.52 μg/mL in four different mediums. In sharp contrast, the equilibrium solubility of DAI in the cocrystal was individually 12.91 ± 1.85, 10.08 ± 2.53, 20.77 ± 0.66 and 295.75 ± 21.80 μg/mL in these mediums. It was found that the formation of a cocrystal resulted in 3.9-, 3.1-, 4.9- and 60.8-fold enhancements in the solubility of DAI in four different media when compared with the parent drug. At the same time, the solubility of DAI in the cocrystal also exhibited a significant increase when compared with the solubility of DAI in the physical mixture.

**Figure 10:**
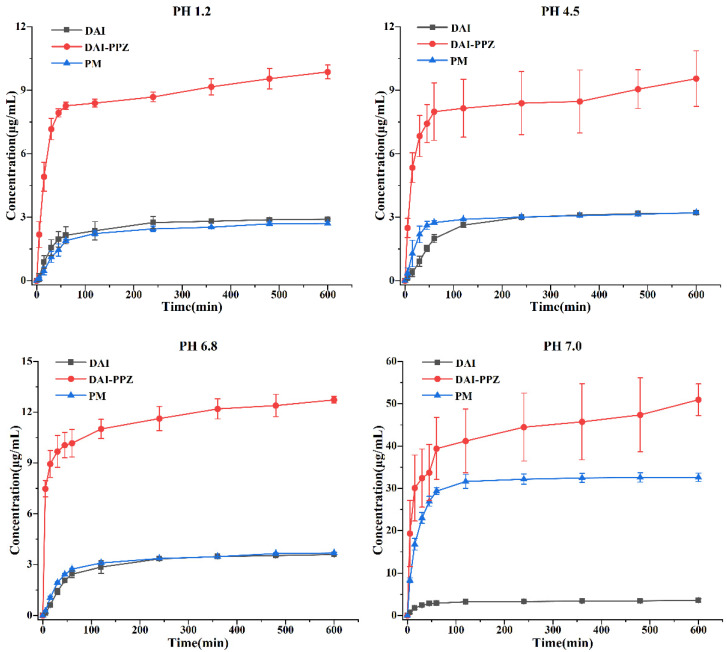
Powder dissolution of the pure DAI, the DAI-PPZ cocrystal and the physical mixture of DAI and PPZ in four different media (pH = 1.2, pH = 4.5, pH = 6.8 and pH = 7.0).

Coupled with the results of equilibrium solubility, the results of powder dissolution in vitro also further confirmed the huge potential of cocrystals in improving the solubility of DAI. It was depicted in Figure 10 that the maximum concentration of pure DAI was separately 2.91 ± 0.04, 3.20 ± 0.05, 3.59 ± 0.10 and 3.59 ± 0.39 μg/mL in four different mediums after 10 h. In the physical mixture, the maximum concentration of DAI was, respectively, 2.71 ± 0.09, 3.21 ± 0.04, 3.68 ± 0.03 and 32.59 ± 0.98 μg/mL in these four mediums after 10 h. In stark contrast, the maximum concentration of DAI in the cocrystal was individually 9.87 ± 0.33, 9.55 ± 1.31, 12.73 ± 0.20 and 50.95 ± 3.74 μg/mL in these four mediums after 10 h. To our surprise, the maximum concentration of DAI in the cocrystal was individually 3.4-, 3.0-, 3.5- and 14.2-fold that of the DAI in these four mediums when it was compared with the pure DAI. Compared with the physical mixture, the maximum concentration of DAI in the cocrystal also achieved significant enhancement in these mediums. In addition, it was obviously found in Figure 10 that the generation of the DAI-PPZ cocrystal also achieved a significant increase in the dissolution rate while elevating the maximum concentration of DAI in these four mediums after 10 h, which could be presented in the comparison of pure DAI, the physical mixture and the DAI-PPZ cocrystal.

By comparing the solution pH values for DAI and DAI-PPZ cocrystal after solubility (Table 3), it was found that the elevation in solubility and dissolution of DAI was associated with an increase in pH value. Based on the previous study [34], DAI showed a significant increase in its solubility under an alkaline environment, which could also be verified by the obvious enhancement of DAI from the physical mixture in distilled water (pH = 7.0). In this study, the DAI-PPZ cocrystal achieved surprising enhancement in the aspect of solubility and dissolution rate, especially in distilled water (pH = 7.0), which might be attributed to the significant changes in pH values in distilled water (6.62 vs. 9.67). This pH-dependent phenomenon of solubility also occurred in some previous reports [35,36]. Apart from the reason for the alkaline environment created by PPZ, some viewpoints should be worthy of deep consideration with respect to the reasons for the optimized solubility of DAI by forming a cocrystal. First, the formation of DAI-PPZ broke the planar structure that DAI itself has. Owing to the formation of DAI-PPZ cocrystal, two phenolic hydroxyl groups were occupied by generating hydrogen bonds (O–H⋯N), which resulted in the impossibility to form intermolecular hydrogen bonds in DAI itself. In addition, PPZ was easily prone to destroying the cocrystal lattice and kept DAI dispersed because of its superior solubility. Furthermore, DAI was not easy to aggregate, and its solubility increases and remains stable without a parachute or spring dissolution behavior with the contribution of the alkaline microenvironment created by PPZ.

### 2.7. Permeability Study

**Figure 11:**
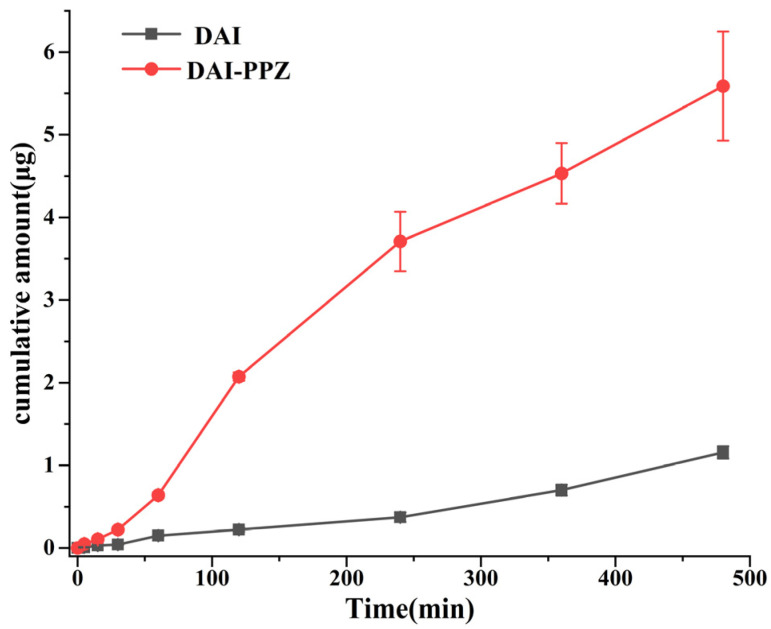
The diffusion profiles of DAI and DAI-PPZ in pH 6.8 buffer media.

To the best of our knowledge, passive diffusion is one of the critical factors affecting drug absorption [37]. In this study, the permeability study of DAI and DAI-PPZ was carried out using a Franz diffusion cell, which provided a relative comparison between DAI and its cocrystal. By comparing the cumulative flux of DAI and DAI-PPZ, the permeability of DAI and DAI-PPZ was evaluated. As depicted in Figure 11, the cumulative flux of DAI reached 1.16 ± 0.08 mg, and the cumulative flux of DAI from DAI-PPZ cocrystal achieved 5.59 ± 0.66 mg. Compared with the pure DAI, the permeability of DAI-PPZ increased 4.8-fold. The results indicated that the generation of DAI-PPZ could significantly optimize the permeability of DAI, which would provide a boost to the bioavailability of DAI. Considering the reason why the cocrystal enhances the permeability of DAI, it might be associated with a higher concentration gradient across the membrane caused by significant elevation in the solubility of DAI, the formation of supramolecular synthons and the molecule interactions between DAI and PPZ, which was consistent with the previous studies [29,37,38,39].

### 2.8. Pharmacokinetics In Vivo

**Figure 12:**
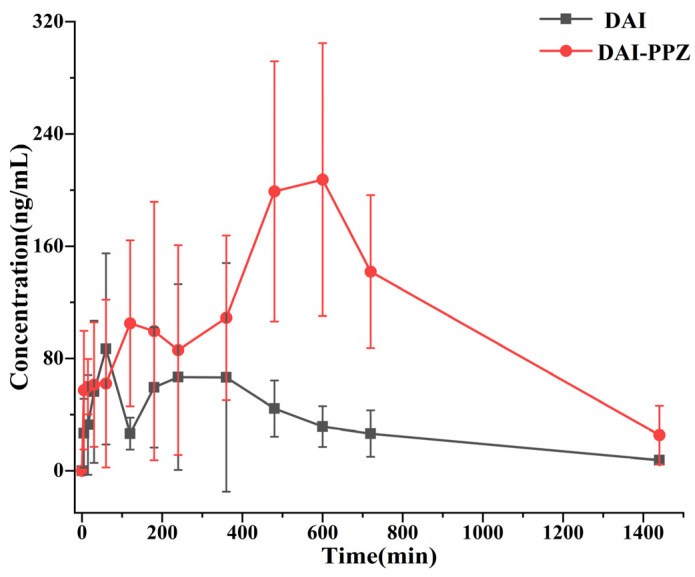
Plasma concentration−time profiles of daidzein and daidzein from the cocrystal after oral administration.

To simulate the delivery process of solid drugs administered orally and prevent changes in the crystalline or cocrystal state of the drug before it enters the stomach, a new kind of solid drug-delivery device was employed to put solid drug powders into the stomachs of rats through their mouths directly in this study. Relying on the high sensitivity, superior accuracy and excellent specificity of HPLC-MS/MS, the plasma concentration of DAI was determined and the plasma concentration–time profiles of DAI-PPZ cocrystal and pure DAI after oral administration were described (Figure 12). Furthermore, the calculated pharmacokinetic parameters are summarized in Table 4. According to the results of pharmacokinetics in rats, the formation of cocrystals arouses a significant change in the pharmacokinetic behaviors of DAI. Compared with the Cmax of pure DAI (131.50 ± 74.29 ng/mL), the Cmax of cocrystal reached 268.47 ± 45.73 ng/mL, which achieved a two-fold boost. By the measurement of AUC_0–∞_, the bioavailability of DAI from cocrystal was 315.4% of that of DAI alone, which fully demonstrated the potential and advantages of DAI-PPZ cocrystal in enhancing the bioavailability of DAI. In addition, delayed T_max_ and increased MRT_0–t_ of DAI from the cocrystal suggested that the generation of the cocrystal prolonged the action time of DAI in vivo. Simultaneously, the significant decrease of CLz/F also reminded us that DAI from the cocrystal possessed a slower rate of elimination in vivo when compared to pure DAI. According to previous reports [7,8,40], high intestine and hepatic metabolism was a main factor influencing the bioavailability of DAI, which would initiate a rapid elimination of DAI. In this study, the application of cocrystal engineering in DAI not only promoted the absorption of DAI but also reduced the elimination of DAI, which facilitated the played efficacy of DAI in vivo. As a whole, the increased bioavailability of DAI with the help of cocrystal technology might be attributed to improved solubility, optimized permeability and attenuated metabolic elimination in vivo.

## 3. Materials and Methods

### 3.1. Materials

In this study, DAI (purity > 98%), genistein (purity > 98%) and PPZ (purity > 99%) were respectively purchased from Wuhan Yuancheng Technology Development Co., Ltd. (Wuhan, China), Shanxi Huike Plant Development Co., Ltd. (Xi’an, China) and Nine-Dinn Chemistry Co., Ltd. (Shanghai, China). All solvents used for crystallization were purchased from the Sinopharm Chemical Reagent Beijing Co., Ltd. (Beijing, China) and their purity belonged to the analytical grade.

### 3.2. Preparation of DAI-PPZ Cocrystal

First, 0.5 mmol of DAI (127.1 mg) and 0.5 mmol of PPZ (43.1 mg) were accurately weighed and placed in a vial. Then, 15 mL of ethanol were added into the vial, and the mixture was stirred at 300 rpm speed for 48 h under the condition of room temperature. Under reduced pressure at 40 °C, the suspension was dried for 72 h to obtain the powder sample of the DAI-PPZ cocrystal.

### 3.3. Preparation of Single Crystal

Using reaction crystallization, the crystal of the DAI-PPZ cocrystal was obtained. At a 1:1 molar ratio, 0.2 mmol of DAI (50.8 mg) and 0.2 mmol of PPZ (17.2 mg) were accurately weighed and placed in a vial. Then, 15 mL of ethanol were added to the vial, and the mixture was stirred at 300 rpm for 48 h under the condition of room temperature. After 48 h, the solution was filtered, and clear and transparent filtrates were obtained. At room temperature, the filtrates were slowly evaporated, and then, colorless block crystals were precipitated after 10 days.

### 3.4. SCXRD Analysis

Performed on a Rigaku XtaLAB Synergy four-circle diffractometer (Rigaku, The Woodlands, TX, USA) with Cu–Kα radiation (wλ = 1.54178 Å), the SCXRD of DAI-PPZ cocrystal was launched at 293 K. Using a direct method and the full-matrix least-squares technique, the structure of a qualified single crystal sample obtained in this study was solved and refined. For the non-hydrogen atoms, its refinement was accomplished using anisotropic displacement parameters. For the hydrogen atoms, they were placed at the calculated positions and refined with a riding model. Using the OLEX2 program (Version 1.5), the SCXRD data of the DAI-PPZ cocrystal were processed and analyzed. In addition, molecular diagrams were obtained with the help of MERCURY software (Version 2023.1.0).

### 3.5. MEPS Calculations

In this study, the MEPS of DAI and PPZ was calculated with the help of DFT. Meanwhile, the B3LYP-D3 function was applied to analyze the process of calculation. In addition, geometric optimizations and single-point energy calculations were achieved with the effort of these two basis sets namely 6-311G (d, p) and 6-311+G (2d, 2p) [41]. The Gaussian 16 package (Version 1.1) was adopted for all calculations, and the Multiwfn 3.7 program was applied for all wave function analyses [42].

### 3.6. PXRD Analysis

In this study, the PXRD experiments were carried out on a Rigaku D/max-2550 diffractometer (Rigaku, Tokyo, Japan). The Cu–Kα radiation source in this diffractometer was set at 40 kV and 150 mA. At a scan rate of 8°/min, the diffraction data were collected from 3 to 40° in the 2θ range. With the help of Jade 6.0 software, the acquired data were analyzed and processed.

### 3.7. IR Analysis

Equipped with an attenuated total reflectance sampling accessory, a PerkinElmer FTIR spectrophotometer (PerkinElmer, Waltham, MA, USA) was applied to conduct the IR experiments. The resolution of the instrument was set as 4 cm^−1^, and 16 scans were performed in the wavelength number range of 4000–400 cm^−1^.

### 3.8. Thermal Analysis

In this study, a Mettler Toledo DSC/DSC 1 (Mettler Toledo, Greifensee, Switzerland) and a Mettler Toledo DSC/TGA1 (Mettler Toledo, Greifensee, Switzerland) were employed to launch thermal analysis. Under a nitrogen gas flow of 50 mL/min, the DSC experiment of DAI, PPZ and their cocrystal was carried out with a heating range from 30 °C to a specified temperature at a constant rate of 10 °C/min. With a heating range of 30–500 °C, the TGA experiment of the DAI-PPZ cocrystal was carried out under a nitrogen gas flow of 50 mL/min at a constant rate of 10 °C/min. In addition, the STAR software package (STARe Default DB V9.10, Mettler Toledo, Greifensee, Switzerland) was adopted to analyze and process the DSC and TG data.

### 3.9. Stability Evaluation

With PXRD analysis, the stability of the DAI-PPZ cocrystal was investigated. In the process of the stability evaluation on the DAI-PPZ cocrystal, 50.0 mg of cocrystal samples were accurately weighed and placed in open containers under three extreme conditions, including high temperature (60 ± 2 °C), high humidity (25 ± 2 °C, 90 ± 5%) and illumination, (4500 ± 500 lx) for 5 and 10 days.

### 3.10. Solubility Determination and PH Measurement

In four different media, including 0.1 M hydrochloric acid aqueous solution (pH = 1.2), acetate buffer (pH = 4.5), phosphate buffer (pH = 6.8) and pure water (pH = 7.0), the equilibrium solubility of DAI, DAI-PPZ cocrystal and the physical mixture of DAI and PPZ was investigated with the saturation shake-flask method. Before the experiment, all samples were sieved through a 100-mesh sieve. An excess amount of samples was added to conical flasks containing 10 mL medium and placed in a ZHWY-103D thermostatic shaker–incubator (Shanghai Zhicheng Analytical Instrument Manufacturing Co., Ltd., Shanghai, China), which were shaken at 200 rpm for 48 h in the air bath at 37 °C. After reaching the equilibrium state, the pH values of all sample solutions were measured using a calibrated pH meter (Mettler Toledo, Zurich, Switzerland). Then, 1 mL of the sample solutions was withdrawn through 0.45 μm nylon filters, and 10 μL of the solutions were injected into the HPLC system to launch the analysis. The HPLC analysis was performed on the Odyssil C18 (4.6 × 250 mm, 5 µm) (Agela Technologies, Wilmington, DE, USA) and the mobile phase consisted of methanol–0.1% acetic acid (70:30, *v*/*v*). In addition, the detection wavelength of DAI was set as 250 nm, and the flow rate was 1.0 mL/min. At the same time, the column temperature was kept at 30 °C, and the injection volume was 10 µL.

### 3.11. Powder Dissolution In Vitro

Before the experiment of powder dissolution in vitro, all samples were sieved through a 100-mesh sieve. Equipped with an automatic sampling system RZQ-12D, an RC12AD dissolution meter (Tianjin TIANDA TIANFA pharmaceutical testing instrument manufacturer, Tianjin, China) was employed to investigate the dissolution of DAI, DAI-PPZ cocrystal and their physical mixture in this study. The 60.0 mg DAI, 80.3 mg DAI-PPZ cocrystal (equivalent to 60.0 mg DAI) and 80.3 mg physical mixtures (the same amount as the cocrystal) were accurately weighed and then added to dissolution vessels filled with 900 mL of four different media, which individually consisted of 0.1 M hydrochloric acid aqueous solution (pH = 1.2), acetate buffer (pH = 4.5), phosphate buffer (pH = 6.8) and pure water (pH = 7.0). In the water bath at 37 °C, samples were stirred at 100 rpm. At 5, 15, 30, 45, 60, 120, 240, 360, 480 and 600 min, 1 mL of sample was collected with an automated sampling system, and 10 μL of the samples were injected into the HPLC.

### 3.12. Flux Measurements

Equipped with a cellulose nitrate membrane (0.45 μm, Cytiva, München, Germany), the modified Franz diffusion cell apparatus was adopted to launch a comparative diffusion study of DAI and DAI-PPZ. Before the experiment, dialysis membranes were mounted in between the donor compartment and the recipient compartment and then activated with pH 6.8 media. The 10 mg of DAI and 13.4 mg of DAI-PPZ cocrystal (equivalent to 10 mg DAI) were accurately weighed and then spread evenly over the membranes. Filled with about 10 mL of pH 6.8 media, the receptor compartment was kept in the water bath at 37 °C. Throughout the experiment, the solution in the receptor compartment was magnetically stirred at 100 rpm. In addition, 0.5 mL of the sample were collected from the receptor compartment at times 5, 15, 30, 60, 120, 240, 360 and 480 min and replaced with the fresh medium at the same time. The collected samples were filtered through 0.45 mm nylon filters, and 10 μL of the samples were injected into HPLC to conduct relative analysis.

### 3.13. Pharmacokinetic Study In Vivo

Supplied by the Experimental Animal Center of the Institute of Materia Medica, Chinese Academy of Medical Sciences, 12 male Sprague–Dawley rats (250 ± 20 g) were housed and handled under suitable humidity, temperature and light. In accordance with the Guideline for Animal Experimentation of the Institute of Materia Medica, Chinese Academy of Medical Sciences, the study related to bioavailability evaluation on the DAI-PPZ cocrystal was carried out. In addition, the protocol was approved by the Animal Ethics Committee of the institution (No. 00009803).

Divided into two groups (*n* = 6 per group) randomly, these rats were individually administrated with DAI (100 mg/kg) or DAI-PPZ cocrystal (133.9 mg/kg, which is equivalent to 100 mg/kg DAI). At 0, 5, 15, 30, 60, 120, 180, 240, 360, 480, 600, 720 and 1440 min after administration, 400 μL blood samples were collected through the retro-orbital venous plexus. After standing for 30 min, the obtained blood samples were centrifuged at 4000 rpm for 10 min at 4 °C. The supernatants were removed from the samples and stored at −80 °C until analysis.

After thawing at room temperature, 100 μL plasma, 20 μL internal standard (genistein, 1.0 μg/mL) solution, 20 μL methanol and 200 μL 0.1 mol/L hydrochloric acid were added to a 1.5 mL Eppendorf tube, respectively. The mixture was vortexed for 1 min, and then, 1.0 mL ethyl acetate was added to it. After vortex-mixing for 3 min, the mixing solution was centrifuged at 13,000 rpm for 10 min. Then, 800 μL of supernatant were withdrawn from the Eppendorf tube and blown to dryness under the nitrogen stream at 40 °C. The residual was redissolved with 100 μL 50% methanol in water and vortexed for 1 min. After centrifugation at 13,000 rpm for 8 min, 3 μL of supernatant were injected into the HPLC/MS/MS for analysis.

Relying on an HPLC system (Shimadzu Corporation, Kyoto, Japan) equipped with a triple quadrupole API 6500+ MS-MS instrument (AB Sciex Instruments, Framingham, MA, USA), the concentration of DAI in plasma was determined accurately. The separation of the substance to be analyzed was performed on a reverse-phase phenomenex HPLC Synergi™ (Torrance, CA, USA) C18 analytical column (50 × 2.0 mm, 4.0 μm) with a mobile phase consisting of 0.1% formic acid in water (solvent A) and acetonitrile (solvent B) at the column temperature of 40 °C. At the same time, the injection volume was 3 μL, and the total flow rate was set as 0.3 mL/min. With a mobile phase ratio of 60:40 (A–B), an isocratic elution procedure was adopted in the process of analysis. The detection of the analytes was achieved in positive mode equipped with a turbo ion spray and electrospray ionization source at 550 °C. In multiple reaction monitoring (MRM) mode, the DAI was analyzed by *m*/*z* 255.1/199.1, and genistein (IS) was analyzed by *m*/*z* 271.1/152.9. After optimization of the mass spectrometry conditions, the declustering potential (DP) of DAI and genistein were individually set as 160 and 140 V, and the collision energy (CE) of DAI and genistein were, respectively, set as 36 and 39 V.

Using analyst software (version 1.6), the data obtained from HPLC/MS/MS was processed. The concentration of DAI in plasma was processed and analyzed with DAS 2.0 software to attain some significant pharmacokinetic parameters. The differences in pharmacokinetic parameters between the two groups were compared and calculated with SPSS 26.0. In addition, the obtained data related to the results were expressed as the mean ± standard deviation (mean ± SD), and the differences between different groups were regarded to be significant statistically when *p* value < 0.05.

## 4. Conclusions

Targeting the dilemma of the poor drug-forming properties of DAI, a novel cocrystal composed of DAI and PPZ at a 1:1 ratio was successfully prepared with the design concept of cocrystal engineering. The formation of the new cocrystal was further characterized and confirmed using various analytical technologies, such as SCXRD, PXRD, IR, DSC and TG. Based on the SCXRD analysis and theoretical calculation, structural details about the interactions in the crystal lattice were clarified and the prepared material was confirmed as a cocrystal. The results of a stability evaluation indicated that the DAI-PPZ cocrystal was stable under high temperature, high humidity and light conditions, which provided a good basis for further development of the cocrystal. By investigating the equilibrium solubility and powder dissolution of DAI-PPZ cocrystal and DAI alone in vitro, it was obviously found that cocrystal technology significantly improved the solubility and dissolution rate of DAI. Compared with the parent drug, the formation of the DAI-PPZ cocrystal, respectively, resulted in a 3.9, 3.1, 4.9 and 60.8-fold enhancement in the solubility of DAI in four different media and achieved a 3.8, 4.0, 4.2 and 13.6-fold increase in the dissolution rate of DAI before the first hour in these four media. The 4.8-fold elevation in the permeability of DAI further demonstrated that cocrystal technology was of significance in enhancing the permeability of DAI. In addition, a 2.0-fold boost in C_max_ and a 3.2-fold increase in the bioavailability of DAI also suggested that DAI-PPZ cocrystal could improve the bioavailability of DAI, which would contribute to enhancing its efficacy. As a simple and effective technique, cocrystal engineering provides a new choice to optimize the pharmaceutical properties of natural products for successful drug formulation and delivery.

## Figures and Tables

**Table 1:** Crystal data and structure refinement parameters of daidzein–piperazine cocrystal.

Parameters	Daidzein–Piperazine Cocrystal
Empirical formula	C_19_H_20_N_2_O_4_
Formula weight	340.37
Crystal size/mm	0.22 × 0.18 × 0.12
Description	block
Crystal system	Monoclinic
Space group	P2_1_/C
a (Å)	12.162(1)
b (Å)	6.385(1)
c (Å)	21.224(1)
β (◦)	95.02(1)
Volume (Å3)	1641.9(1)
Z	4
Completeness	99.7%
Density (g·cm^−3^)	1.377
Reflections with I > 2σ (I)	2792
Rindexs (I > 2σI)	R1 = 0.0556, wR2 = 0.1578
Goodness of fit on F2	1.035
CCDC deposition number	2313133

**Table 2:** Hydrogen bonds of daidzein–piperazine cocrystal.

D–H⋯A	d(D⋯A) (Å)	∠(DHA) (deg)	Symmetry Code
O4−H4⋯N2P	2.738	146.91	-
O3−H3⋯N1P	2.557	131.51	[x + 1, −y + 1/2, z − 1/2]
N1P−H1P⋯O2	2.919	127.93	[−x, −y + 1, −z + 1]
N2P−H2P⋯O3	3.092	175.17	[−x + 1, −y + 1, −z + 1]

**Table 3:** The solution pH values for DAI, DAI-PPZ cocrystal and their physical mixture (PM) after solubility (mean ± SD, *n* = 3).

Compound	PH 1.2	PH 4.5	PH 6.8	PH 7.0
DAI	1.31 ± 0.06	4.72 ± 0.01	6.80 ± 0.04	6.62 ± 0.57
DAI-PPZ	1.80 ± 0.35	4.86 ± 0.01	7.23 ± 0.05	9.67 ± 0.21
PM	1.88 ± 0.01	4.82 ± 0.02	7.18 ± 0.07	9.70 ± 0.17

**Table 4:** Pharmacokinetic parameters after oral administration of daidzein and daidzein–piperazine cocrystal in rats (x ± s, *n* = 6).

Parameter	Daidzein	Daidzein-Piperazine
AUC(0–t) (µg/L·h)	798.15 ± 398.10	2600.55 ± 701.41 **
AUC(0–∞) (µg/L·h)	895.53 ± 388.94	2824.12 ± 764.66 **
MRT(0–t) (h)	7.89 ± 1.04	9.79 ± 1.14 *
MRT(0–∞) (h)	12.29 ± 5.50	11.61 ± 3.04
t1/2z (h)	8.58 ± 6.16	4.76 ± 2.18
T_max_ (h)	3.75 ± 2.89	8.17 ± 2.71 *
CLz/F (L/h/kg)	128.09 ± 47.35	50.57 ± 14.12 **
C_max_ (µg/L)	131.50 ± 74.29	268.47 ± 45.73 **

Notes: ** *p* < 0.01 between daidzein and daidzein–piperazine cocrystal, * *p* < 0.05 between daidzein and daidzein–piperazine cocrystal.

## Data Availability

The data on the compounds are available from the authors.
